# Knowledge, attitude, and practice toward tuberculosis prevention and management among household contacts in Suzhou Hospital, Jiangsu province, China

**DOI:** 10.3389/fpubh.2024.1249971

**Published:** 2024-03-12

**Authors:** Yuping Zhang, Jingwen Wu, Xiaoyan Hui, Peipei Zhang, Fang Xue

**Affiliations:** Department of Infectious Disease, Suzhou Hospital of Integrated Traditional Chinese and Western Medicine, Suzhou, China

**Keywords:** tuberculosis, household contacts, prevention, management, knowledge, attitude, practice

## Abstract

**Background:**

China is among the 10 high-burden tuberculosis (TB) countries in the world; thus, investigation and management of household contacts is an essential part of TB prevention strategy.

**Objective:**

To explore the knowledge, attitude, and practice (KAP) toward TB prevention and management among household contacts of TB patients.

**Methods:**

This cross-sectional study enrolled household contacts in Suzhou Hospital of Integrated Traditional Chinese and Western Medicine between September 2022 and January 2023; KAP and demographic characteristics were assessed with the self-designed questionnaire and analyzed by multivariate logistic regression.

**Results:**

A total of 503 participants were included; of them, 280 (55.78%) were female, and 303, (60.36%) aged ≥45 years. The KAP scores were 6.24 ± 2.20 (possible range: 0–12), 18.69 ± 2.80 (possible range: 0–36), and 20.37 ± 5.15 (possible range: 0–36), respectively. Suburban (OR = 0.18, 95% CI: 0.04–0.79, *p* = 0.023) and rural (OR = 0.12, 95% CI: 0.03–0.57, *p* = 0.008) were independently associated with knowledge. Positive attitude (OR = 7.03, 95% CI: 2.92–16.96, *p* < 0.001), education (high school or technical secondary school, OR = 4.91, 95% CI: 1.63–14.73, *p* = 0.005; college and above, OR = 14.94, 95% CI: 3.51–63.58, *p* < 0.001), and shorter disease duration (3–6 months, OR = 0.40, 95% CI: 0.18–0.90, *p* = 0.026) were independently associated with better practice scores.

**Conclusion:**

Household contacts of TB patients demonstrated insufficient knowledge, unfavorable attitude, and suboptimal practice toward TB prevention and management. Tailored interventions are needed to ensure information accessibility, especially for individuals living in suburban and rural areas.

## Introduction

Tuberculosis (TB) is a communicable disease, which is caused by mycobacterium tuberculosis (1, 2). In 2021, an estimated 10.6 million people contracted TB, and 1.6 million died from the disease (1). In 2014 and 2015, the World Health Organization (WHO) and the United Nations (UN) adopted WHO’s end TB Strategy, aiming to eliminate the global TB epidemic by the year 2035 (3). China is one of the highest TB burden countries, with a total TB incidence of 55 per 100,000 and approximately 6,395,000 new tuberculosis cases in 2021 (4). It was estimated that China had the highest burden of latent tuberculosis infection (LTBI) in the world, with approximately 350 million persons living with the infection (5). Household contacts of TB patients are at a higher risk of developing TB due to prolonged and close exposure to source (6), with a prevalence of 5.2% in those ≥15 years old (7). Therefore, investigation and management of household contacts are discussed as essential parts of the TB prevention strategy (1, 8).

Knowledge, attitude, and practice (KAP) study is a structured method that provides access to quantitative and qualitative information regarding a known situation, might reveal misunderstandings or potential barriers to the implementation of healthcare practices, identify what is known and done about various health-related subjects, and plan new interventions (9, 10). It was demonstrated that the methodology of the KAP study could provide useful insight for TB management, as lower KAP scores were associated with delayed healthcare seeking (11). The majority of previous studies accessed KAP toward TB among healthcare workers or TB patients, reporting mostly encouraging results (2, 12–14). However, a recent study undertaken in India has found that compared to TB patients, household contacts had notably poorer knowledge (15). Based on that, KAP of TB prevention and management among household contacts needs to be further studied to develop effective interventions. In addition, this significance is amplified by the distinctive living arrangements found in Chinese families, where at least three generations live together and share the same spaces, making it imperative to gain insights into these dynamics (16).

Although China has made some progress in reducing TB cases, it is still far from achieving the goals of the end TB strategy. A study published in 2021 (17) reported the notable absence of tuberculosis-related training for health professionals and the lower efficacy of primary healthcare providers in referring and managing TB cases among the main healthcare challenges. In 2019, the State Council of China issued the new Healthy China Initiative 2019–2030, which included the Tuberculosis Control Action focused on patient-centered approach with greater attention to vulnerable and hard-to-reach populations (18). Moreover, in the most recent study conducted in Shanghai, China, close contact investigation proved to be at least partly effective strategy for TB control (19). Therefore, this study aimed to explore the KAP toward TB prevention and management among household contacts of TB patients in China. We hypothesized that practice patterns might be related to knowledge and/or attitude, and additional modifiable factors could be identified to plan specific educational interventions in the target population.

## Materials and methods

### Study design and participants

This cross-sectional quantitative study enrolled household contacts in Suzhou Hospital of Integrated Traditional Chinese and Western Medicine between September 2022 and January 2023. Inclusion criteria are as follows: (1) age ≥18 years and (2) living with TB patients for more than 7 days during the period between 3 months before the diagnosis of TB and 14 days after the start of treatment of TB. Exclusion criteria are as follows: (1) cognitive dysfunction and (2) combined serious life-threatening diseases. A maximum of two family members per patient were included in the survey. The study has been ethically approved by the Ethics Committee of Suzhou Hospital of Integrated Traditional Chinese and Western Medicine (2022-001), and informed consent was obtained from the study participants.

### Questionnaire and quality control

The questionnaire was designed with reference to the related literature (2, 20, 21), and WHO guidelines on tuberculosis infection prevention and control: 2019 update, the Technical Guidelines for the Prevention and Management of Tuberculosis in China (22, 23), and the Guidelines for Primary Care of Tuberculosis (Expert Consensus) (24). The first draft was modified by comments from two experts. A pretest was carried out (*n* = 59), and Cronbach’s *α* was 0.829, indicating a good internal consistency.

The final questionnaire included four sections with 43 items in total (Supplementary material). The demographic characteristics included 12 items; the knowledge section included 11 items; the attitude section included 10 items; and the practice section included 10 items. For knowledge items, K1–10 scored 1 point for each correct answer, 0 points for a wrong answer or unclear; K11 was about the risk factors of TB, with “well informed,” “partially know,” and “do not know” were scored 2, 1, and 0, the knowledge scores ranging from 0 to 12 points. The attitude items were scored ranging from extremely positive (4 points) to extremely negative (0 points), while items A3 were not scored due to the lack of a clear positive or negative tendency; thus, the possible score range of attitude was 0–36 points. For the practice items, P1, P3, and P4, the presence of positive behavior was assigned 4 points, and the absence of it was assigned 0 points; for P2, the presence of negative behavior was assigned 0 points, and the absence of it was assigned 4 points; P5–9 were scored according to the perspective of positive behavior, ranging from “totally compliantly” (4 points) to “not compliantly at all” (0 points); P10 was to investigate the access to knowledge, which could not be assigned a score. Thus, the score range of the practice was also 0–36 points. According to Blooms’ cut-off point (25), knowledge, attitude, and practice scores of 60% or more of the theoretical total score were considered as “adequate knowledge” (≥7.2), “positive attitude” (≥21.6), and “proactive practice” (≥21.6).

The possibility of participating in the study was discussed with pulmonary tuberculosis patients who were newly diagnosed during the study period. In addition to this, researchers presented the study to previously diagnosed patients who come to the hospital for follow-up visits. Upon agreeing, TB patients received letters from their family members with explanations and invitations to the study site to answer questionnaires. If the family member was present during the visit, they were invited to discuss the informed consent form and fill in the questionnaire right away. Before paper-based questionnaires were distributed in the consultation room, the research team assured participants that there were no right or wrong answers and encouraged them to truthfully fill out the questionnaire. Additionally, the team offered prompt assistance if any difficulties arose during the filling process. Subsequently, a statistician conducted quality control measures, in which any questionnaires that exhibited obvious logical errors or consistently selected the same option throughout were identified as invalid and excluded from the analysis. This rigorous process aimed to uphold the quality and validity of the data collected from the participants, enhancing the credibility of the study’s findings.

### Sample size calculation

The sample size was calculated based on the item-respondent theory (26). A ratio of 1:10 is considered suitable according to this theory. Considering 31 KAP items of the questionnaire, the required sample size was 310. Considering a 20% drop-out rate, the final sample size was 388.

### Statistical analysis

Stata 17.0 (Stata Corporation, College Station, TX, United States) was used for statistical analysis. The continuous variables with normal distribution were presented as means ± standard deviations (SD) and tested using an independent sample *t*-test or one-way analysis of variance (ANOVA). Continuous variables with skew distribution were presented as *n* (percentage) and tested using the Wilcoxon–Mann–Whitney test or Kruskal–Wallis analysis of variance. Categorical variables were presented as *n* (percentage). Pearson’s correlation tests were used to analyze the correlation between knowledge, attitude, and practice. Variables with *p* < 0.05 in the univariate logistic regression were included in the multivariate logistic regression. Multivariate logistic regression was conducted to determine the factors associated with KAP. A two-sided *p* < 0.05 was considered statistically significant.

## Results

A total of 503 questionnaires were collected, and one of them was excluded due to obvious repetition of the same option, leaving 502 valid questionnaires (99.80%). Among the participants, 303 (60.36%) were aged ≥45 years old, 280 (55.78%) were females, 448 (89.24%) were married, and 210 (41.83%) were living in rural areas. For the relationships with TB patients, 212 (42.23%) and 146 (29.08%) were parents and spouses, respectively. Over half of the patients in their family were < 6 months after first diagnosed with TB (51.00%) and in initial treatment (78.49%) (Table 1).

**Table 1:** Demographic characteristics and KAP score among household contacts of TB.

Variables	*N* (%)	Knowledge scores		Attitude scores		Practice scores	
		Mean ± SD	*p*	Mean ± SD	*p*	Mean ± SD	*p*
Total	502	6.24 ± 2.20		18.69 ± 2.80		20.37 ± 5.15	
Sex			0.107		0.880		0.499
Male	222 (44.22)	6.41 ± 2.15		18.72 ± 2.83		20.27 ± 5.38	
Female	280 (55.78)	6.11 ± 2.23		18.66 ± 2.78		20.45 ± 4.97	
Age, years			<0.001		0.305		<0.001
≤44	199 (39.64)	7.01 ± 2.24		18.96 ± 2.79		21.57 ± 5.48	
45–59	215 (42.83)	6.15 ± 1.92		18.45 ± 2.68		20.25 ± 4.77	
≥60	88 (17.53)	4.72 ± 1.90		18.63 ± 3.09		17.97 ± 4.40	
Marital status			0.117		0.068		0.369
Married	448 (89.24)	5.76 ± 2.39		19.43 ± 2.76		20.54 ± 5.81	
Unmarried/divorced/widowed	54 (10.76)	6.30 ± 2.17		18.60 ± 2.80		20.35 ± 5.07	
Residence			<0.001		<0.001		<0.001
Rural	210 (41.83)	5.44 ± 2.19		18.09 ± 2.68		18.62 ± 4.64	
Urban	21 (4.18)	7.90 ± 1.92		20.86 ± 4.25		24.81 ± 4.70	
Suburban	271 (53.98)	6.73 ± 2.00		18.98 ± 2.64		21.39 ± 5.10	
Education level			<0.001		<0.001		<0.001
Primary school and below	98 (19.52)	4.23 ± 1.77		17.97 ± 2.75		16.60 ± 3.36	
Middle school	218 (43.43)	6.07 ± 1.69		18.48 ± 2.67		19.94 ± 4.69	
High school/technical secondary school	107 (21.31)	6.98 ± 2.18		18.76 ± 2.61		21.18 ± 4.97	
College and above	79 (15.74)	8.19 ± 1.73		20.04 ± 3.04		25.16 ± 4.36	
Work status			<0.001		0.012		0.273
Employed	424 (84.46)	6.44 ± 2.14		18.54 ± 2.70		20.45 ± 5.07	
Unemployed	78 (15.54)	5.18 ± 2.22		19.46 ± 3.19		19.97 ± 5.61	
Monthly family income *per capita* (CNY)			<0.001		0.002		<0.001
<2,000	35 (6.97)	3.94 ± 1.76		18.60 ± 2.98		16.11 ± 3.27	
2,000–4,999	413 (82.27)	6.07 ± 1.97		18.53 ± 2.75		20.05 ± 4.90	
5,000–9,999	54 (10.76)	9.07 ± 1.21		19.91 ± 2.82		25.65 ± 3.99	
Medical insurance			<0.001		0.237		<0.001
Yes	482 (96.02)	6.36 ± 2.13		18.71 ± 2.82		20.59 ± 5.11	
No	20 (3.98)	3.30 ± 1.72		18.15 ± 2.41		15.10 ± 2.85	
Relationship with the patient			<0.001		0.036		<0.001
Spouse	146 (29.08)	6.91 ± 1.81		19.26 ± 3.25		21.05 ± 5.75	
Parents	212 (42.23)	5.34 ± 1.93		18.25 ± 2.47		19.01 ± 4.30	
Children/grandchildren and their spouses	129 (25.70)	7.15 ± 2.40		18.71 ± 2.64		22.01 ± 5.15	
Sibling	15 (2.99)	4.67 ± 1.63		19.00 ± 3.00		18.93 ± 5.19	
Disease duration of the patient			0.037		<0.001		<0.001
<3 months	167 (33.27)	6.13 ± 2.28		19.55 ± 2.48		21.20 ± 5.60	
3–6 months	89 (17.73)	5.96 ± 2.18		19.51 ± 2.65		20.54 ± 5.81	
6 months to 1 year	152 (30.28)	6.71 ± 2.09		18.81 ± 2.61		20.64 ± 4.61	
≥1 year	94 (18.73)	5.96 ± 2.14		16.17 ± 2.28		18.32 ± 3.87	
Most recent smear result of the patient			<0.001		<0.001		<0.001
Positive	156 (31.08)	6.03 ± 2.32		18.89 ± 2.72		20.96 ± 5.49	
Negative	291 (57.97)	6.58 ± 2.07		18.88 ± 2.80		20.72 ± 4.86	
Unclear	55 (10.96)	5.02 ± 2.02		17.05 ± 2.54		16.91 ± 4.37	
Treatment status of the patient			0.001		<0.001		<0.001
Initial treatment	394 (78.49)	6.40 ± 2.21		19.32 ± 2.57		21.19 ± 5.20	
Retreatment	45 (8.96)	5.89 ± 2.39		15.84 ± 2.14		18.07 ± 3.41	
Unknown	63 (12.55)	5.49 ± 1.80		16.76 ± 2.49		16.92 ± 3.80	

**Figure 1:**
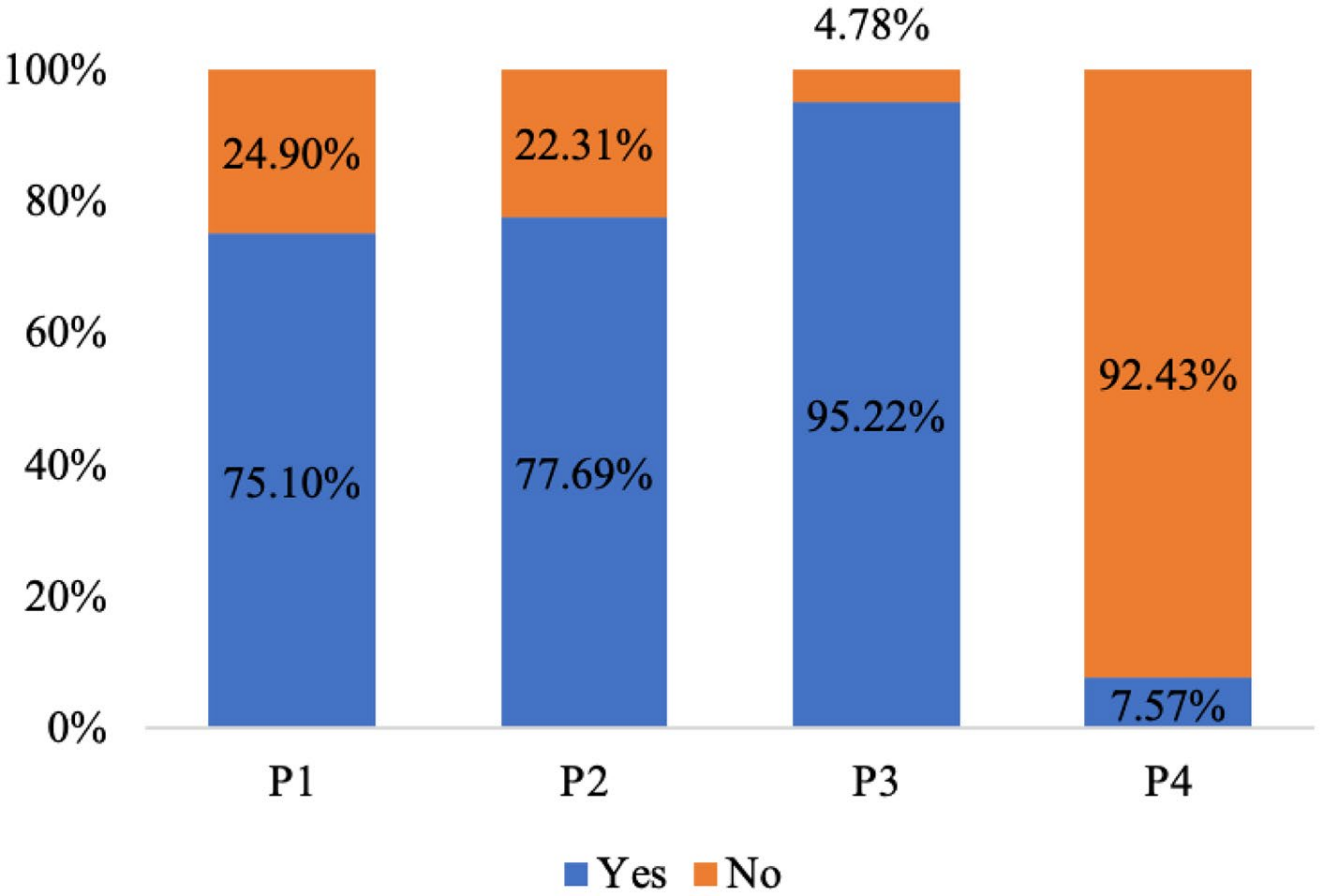
Practice of TB household contacts. P1. The TB patient in your household lives alone in one room. P2. You are still eating with the TB patient in your household after he/she was diagnosed. P3. Did you go to the hospital for TB screening after the patient was diagnosed? P4. Did you receive preventive treatment?

**Figure 2:**
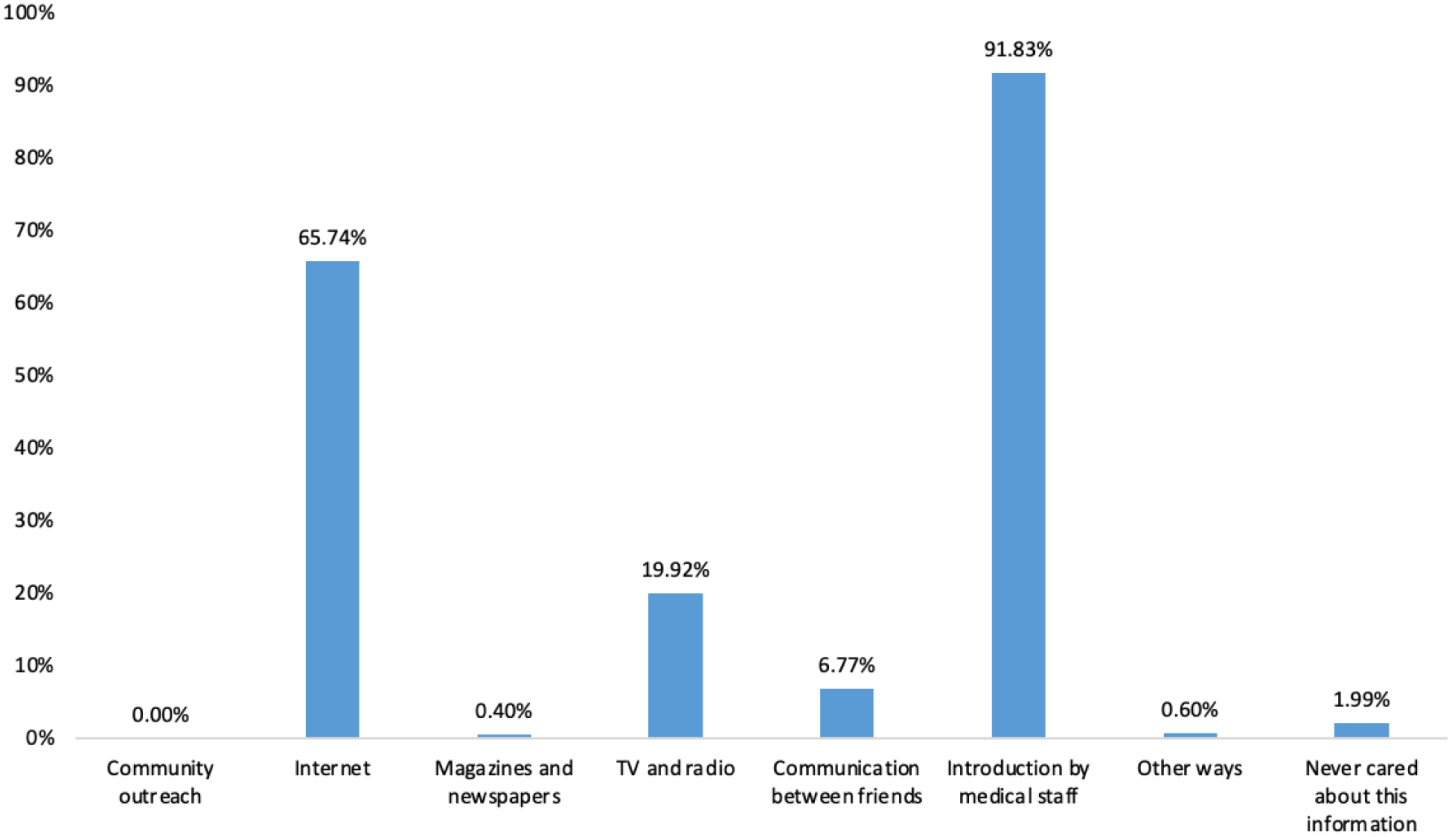
Access to the knowledge of TB among TB household contacts.

The scores of knowledge, attitude, and practice were 6.24 ± 2.20 (possible range: 0–12), 18.69 ± 2.80 (possible range: 0–36), and 20.37 ± 5.15 (possible range: 0–36), respectively. The knowledge, attitude, and practice scores were all varied among different education, family *per capita* income, relationship with the patient, and disease duration of the patient in their family (all *p* < 0.05) (Table 1). The knowledge item with the highest correct rate was “TB is mainly transmitted through the respiratory tract, such as droplets and dust” (K2), with a correct rate of 96.41%; the question with the lowest correct rate was “People with latent TB infection are not TB patients and are not infectious” (K3), with a correct rate of 10.36% (Table 2). For the attitude items, the highest rate of agree/strongly agree item was “You can get along with TB patients as an equal” (A6), with a rate of 91.04%; the item with the lowest rate of agree/strongly agree was “Would you agree to preventative treatment for close contacts in families with children under 5 years old?” (A10), with the rates of 2.39% (Table 3). Regarding the practice items, 95.22% of the participants went to hospitals for TB screening after the patient was diagnosed, and only 7.57% of the participants received preventive treatments (P3), 92.4% presented negative behavior of eating with the TB patient after he/she was diagnosed (P4) (Figure 1). Almost 70.72% of participants reported that they would pay attention to opening windows for ventilation air, washing hands frequently, and taking active physical exercise (P8). However, only 37.45% of participants reported that they would try their best to avoid direct contact with patients (P6) (Table 4). The main access for TB household contacts to learn about TB were media staff (91.83%), internet (65.74%), and TV and radios (19.92%) (Figure 2).

**Table 2:** Knowledge.

Knowledge	Correct/well informed, *n* (%)
1. Tuberculosis (TB) is a chronic infectious disease caused by mycobacterium TB	478 (95.22)
2. TB is mainly transmitted through the respiratory tract, such as droplets and dust	484 (96.41)
3. People with latent TB infection are not TB patients and are not infectious	52 (10.36)
4. Coughing for longer than 2 weeks or hemoptysis are common suspicious symptoms of TB; prompt consultation and treatment are needed	432 (86.06)
5. The regular medication treatment course for TB patients should be at least 6 months and be adjusted accordingly by considering the condition of TB and drug resistance	373 (74.30)
6. The patient can stop taking medications when the symptoms disappear during the treatment of TB	397 (79.08)
7. The close contacts with negative results of TB screening test should be screened again after half a year and 1 year	123 (24.50)
8. BCG vaccination can prevent children from TB infection	337 (67.13)
9. Disinfection can be achieved by directly drying the articles used by patients in strong sunlight for half an hour	77 (15.34)
10. Boiling and high-pressure steam disinfection are the most effective methods to kill tuberculous bacteria and should continue to boil for more than 10 min	280 (55.78)
11. Risk factors for TB: HIV infection, history of tuberculosis exposure, immune weakness, etc	96 (19.13)

**Table 3:** Attitude.

Items	Strongly agree, *n* (%)	Agree, *n* (%)	Neutral, *n* (%)	Disagree, *n* (%)	Strongly disagree, *n* (%)
A1. TB is preventable and treatable	81 (16.14)	278 (55.38)	130 (25.90)	13 (2.59)	0
A2. You Are worried about being infected with TB	31 (6.18)	160 (31.87)	46 (9.16)	257 (51.20)	8 (1.59)
A3. The occurrence of TB patients in your family has a great impact on your life^a^	174 (34.66)	288 (57.37)	21 (4.18)	16 (3.19)	3 (0.60)
A4. You are ashamed of having TB patients in your family^b^	56 (11.16)	222 (44.22)	70 (13.94)	139 (27.69)	15 (2.99)
A5. You do not want others to know that there are TB patients in your family^b^	135 (26.89)	319 (63.55)	42 (8.37)	5 (1.00)	1 (0.20)
A6. You can interact with tuberculosis patients with an attitude of equality and understanding	228 (45.42)	229 (45.62)	45 (8.96)	0	0
A7. TB patients and their household contacts will be discriminated by people around them^b^	37 (7.37)	399 (79.48)	46 (9.16)	18 (3.59)	2 (0.40)
A8. You wish to get more information about TB prevention and treatment	19 (3.78)	388 (77.29)	95 (18.92)	0	0
A9. You are willing to receive preventive treatment if your doctor recommends it	35 (6.97)	185 (36.85)	271 (53.98)	11 (2.19)	0
A10. Would you agree to preventative treatment for close contacts in families with children under 5 years old?	5 (1.00)	7 (1.39)	284 (56.57)	206 (41.04)	0

a Item that is not assigned a score.

b Item that is reversed assigned.

**Table 4:** Practice toward TB among household contacts.

Practice items	Totally compliantly, *n* (%)	Compliantly, *n* (%)	Moderate, *n* (%)	Not compliantly, *n* (%)	Not compliantly at all, *n* (%)
P5. You will supervise the patient to take medications on time according to the doctor’s advice	57 (11.35)	233 (46.41)	146 (29.08)	66 (13.15)	0
P6. You will try your best to avoid direct contact with patients	12 (2.39)	176 (35.06)	57 (11.35)	239 (47.61)	18 (3.59)
P7. You will remind patients and other family members to cover their mouths and noses with tissues when sneezing or coughing	39 (7.77)	237 (47.21)	196 (39.04)	30 (5.98)	0
P8. You will pay attention to opening windows for ventilation air, washing hands frequently, and taking active physical exercise	43 (8.57)	312 (62.15)	138 (27.49)	9 (1.79)	0
P9. You will take the initiative to learn about TB	19 (3.78)	294 (58.57)	165 (32.87)	24 (4.78)	0

Knowledge was positively correlated with attitude (*r* = 0.223, *p* < 0.001) and practice (*r* = 0.539, *p* < 0.001), and attitude was positively correlated with practice (*r* = 0.379, *p* < 0.001) (Table 5). Suburban (OR = 0.18, 95% CI: 0.04–0.79, *p* = 0.023) and rural (OR = 0.12, 95% CI: 0.03–0.57, *p* = 0.008) were independently associated with knowledge. College and above (OR = 5.28, 95% CI: 1.55–17.99, *p* = 0.008) was associated with attitude. Positive attitude (OR = 7.03, 95% CI: 2.92–16.96, *p* < 0.001), high school or technical secondary school (OR = 4.91, 95% CI: 1.63–14.73, *p* = 0.005), and college and above (OR = 14.94, 95% CI: 3.51–63.58, *p* < 0.001), monthly income of 5,000–9,999 CNY (OR = 14.26, 95% CI: 2.04–99.44, *p* = 0.007) and disease duration of the patients with 3–6 months (OR = 0.40, 95% CI: 0.18–0.90, *p* = 0.026) were associated with practice (Table 6).

**Table 5:** Correlations between knowledge, attitude, and practice.

Variables	Knowledge	Attitude	Practice
Knowledge	1		
Attitude	0.223 (*p* < 0.001)	1	
Practice	0.539 (*p* < 0.001)	0.379 (*p* < 0.001)	1

**Table 6:** Multivariate logistic regression.

Variables	OR (95% CI)	*p*
**Knowledge**		
*Age*		
≤44	Ref.	
45–59	1.15 (0.50, 2.65)	0.738
≥60	1.63 (0.42, 6.29)	0.480
*Residence*		
Urban	Ref.	
Rural	0.12 (0.03, 0.57)	0.008
Suburban	0.18 (0.04, 0.79)	0.023
*Education*		
Primary school and below	Ref.	
Middle school	9.51 (1.79, 50.62)	0.008
High school/technical secondary school	35.19 (5.55, 223.07)	<0.001
College and above	49.39 (6.82, 357.56)	<0.001
*Work status*		
Employed	Ref.	
Unemployed	0.48 (0.18, 1.31)	0.152
*Monthly family income per capita (CNY)*		
<2,000	Ref.	
2,000–4,999	0.74 (0.13, 4.15)	0.733
5,000–9,999	6.04 (0.80, 45.49)	0.081
*Relationship with the patient*		
Spouse	Ref.	
Parents	0.59 (0.27, 1.28)	0.182
Children/grandchildren and their spouses	0.78 (0.40, 1.51)	0.457
Siblings	*Omitted*	
*Most recent smear result of the patient*		
Negative	Ref.	
Positive	0.50 (0.28, 0.89)	0.019
Unclear	1.10 (0.35, 3.44)	0.865
*Treatment status of the patient*		
Initial treatment	Ref.	
Retreatment	1.54 (0.63, 3.79)	0.342
Unknown	0.31 (0.11, 0.93)	0.036
**Attitude**		
*Knowledge*		
[0, 7.2]	Ref.	
[7.20, 12]	1.12 (0.56, 2.21)	0.753
*Residence*		
Urban	Ref.	
Rural	0.59 (0.17, 2.01)	0.401
Suburban	0.84 (0.27, 2.59)	0.763
*Education*		
Primary school and below	Ref.	
Middle school	1.67 (0.66, 4.24)	0.277
High school/technical secondary school	1.31 (0.40, 4.30)	0.658
College and above	5.28 (1.55, 17.99)	0.008
*Work status*		
Employed	Ref.	
Unemployed	1.95 (0.90, 4.21)	0.089
*Relationship with the patient*		
Spouse	Ref.	
Parents	0.49 (0.24, 0.99)	0.048
Children/grandchildren and their spouses	0.24 (0.11, 0.55)	<0.001
Siblings	0.59 (0.11, 3.11)	0.534
*Disease duration of the patient*		
<3 months	Ref.	
3–6 months	0.98 (0.49, 1.94)	0.950
6 months to 1 year	0.57 (0.29, 1.11)	0.098
≥1 year	0.38 (0.04, 3.81)	0.408
*Treatment status of the patient*		
Initial treatment	Ref.	
Retreatment	0.26 (0.01, 5.34)	0.379
Unknown	0.17 (0.02, 1.62)	0.123
**Practice**		
*Knowledge*		
[0, 7.2]	Ref.	
[7.20, 12]	1.45 (0.79, 2.64)	0.231
*Attitude*		
[0, 21.60]	Ref.	
[21.60, 36]	7.03 (2.92, 16.96)	< 0.001
*Age*		
≤44	Ref.	
45–59	1.54 (0.69, 3.46)	0.294
≥60	0.98 (0.34, 2.77)	0.963
*Residence*		
Urban	Ref.	
Rural	0.36 (0.08, 1.66)	0.189
Suburban	0.38 (0.08, 1.75)	0.216
*Education level*		
Primary school and below	Ref.	
Middle school	1.92 (0.94, 3.91)	0.072
High school/technical secondary school	4.91 (1.63, 14.73)	0.005
College and above	14.94 (3.51, 63.58)	<0.001
*Monthly family income per capita (CNY)*		
<2,000	Ref.	
2,000–4,999	2.71 (0.89, 8.31)	0.081
5,000–9,999	14.26 (2.04, 99.44)	0.007
*Relationship with the patient*		
Spouse	Ref.	
Parents	1.32 (0.71, 2.45)	0.379
Children/grandchildren and their spouses	0.83 (0.42, 1.64)	0.591
Siblings	0.48 (0.14, 1.71)	0.259
*Disease duration of the patient*		
<3 months	Ref.	
3–6 months	0.40 (0.18, 0.90)	0.026
6 months to 1 year	0.90 (0.43, 1.89)	0.778
≥1 year	2.23 (0.57, 8.72)	0.248
*Most recent smear result of the patient*		
Negative	Ref.	
Positive	0.64 (0.33, 1.24)	0.186
Unclear	0.79 (0.35, 1.82)	0.586
*Treatment status of the patient*		
Initial treatment	Ref.	
Retreatment	0.15 (0.04, 0.62)	0.009
Unknown	0.11 (0.03, 0.36)	<0.001

It also showed differences in knowledge, attitude, and practice dimensions across residences (Supplementary Table S1). For knowledge items, the scores for basic knowledge of TB, TB treatment-related knowledge, and contacts’ knowledge of TB prevention were significantly different among participants from urban, rural and suburban areas (all *p* < 0.05).

## Discussion

This study found insufficient knowledge, unfavorable attitude, and suboptimal practice toward TB prevention and management among household contacts of TB patients in China. The majority of preventive measures violations, such as 92.4% of participants eating together with the TB patient even before the treatment started and 37.45% not trying to avoid direct contact with patients, were most probably explained by the inability to change living arrangements. In-hospital education was identified as a primary (and sometimes only) source of information regarding TB management. These results might help the establishment of prevention measures for the promotion of healthy lifestyles and TB management among household contacts.

A few previous studies undertaken among household contacts of TB patients reported varied results, from poor knowledge, but positive attitude in India (15) to acceptable knowledge/attitude with poor practice in Malaysia (27). This disparity could be attributed to variations in racial, geographical, and cultural characteristics of the populations studied, as well as the differences in the approach to the questionnaire. Nevertheless, some common points were also demonstrated, such as the fact that good knowledge, positive attitude, and proactive practice toward TB were significantly higher among those with higher education, which is also consistent with studies undertaken among TB patients (11), general population (12), university students (28), and social media users (29). Another common observation is the difference between urban and rural populations, with urban residents more likely to have adequate knowledge, favorable attitudes, or good practices toward TB (29–31). In the present study In the present study, knowledge scores itself were notably higher in urban residents compared to rural (7.90 ± 1.92 vs. 5.44 ± 2.19, *p* < 0.001). Although the percentage of urban residents in our study was low, due to the generally lower prevalence, suburban and rural residence was still independently associated with lower knowledge scores. It necessitates the clear need for educational interventions designed and implemented specifically for rural and suburban areas to facilitate a better understanding of tuberculosis prevention and management.

Violations of TM prevention measures, found in the present study, are most likely equally preconditioned by lower education and income levels of participants, higher number of people living in the same household, and their physical inability to change living arrangements; those features are somewhat typical for rural China, calling for integral systematic changes at all levels (32, 33). However, some improvements might be possible to implement on a local plane. For one thing, in this study, comparing to spouses of the TB patients, the attitude score of their parents, children, and grandchildren was significantly lower; it suggests that the relationship with the patient could play a motivating role in education and tailored intervention might improve the attitude of the household contacts with different family relationships, resulting in the better practice. Aside from that, in-hospital education was identified as a primary source of information for study participants, sometimes being the only source. The results also found that knowledge, attitude, and practice were positively related to each other, consistent with the notion that adequate knowledge is associated with good practices among general populations. Thus, this study emphasizes the importance of effectively communicating essential information to household contacts during hospital visits and even stronger need for alternative ways to reach family members who are unable to visit the hospital. Efforts made to improve KAP toward TB should prioritize individuals with lower education levels, those with a lower socio-economic status, and members of the family other than their spouse.

This study has some limitations. First of all, it is a single-center study with limited sample size. Most importantly, the study was undertaken in the hospital and thus excluded family members who were unable to commute. Second, self-reported questionnaires were used for the measurement of KAP, which may produce information bias. Third, there may be some confounding factors, for example, the KAP of the TB patients, and the health education practice provided by healthcare providers, which were not measured in this study. Despite the limitations, the findings might provide implications for the development of TB education programs and intervention strategies for household contacts of TB patients in China.

In conclusion, this study demonstrated insufficient knowledge, unsatisfactory attitude, and suboptimal practice toward TB prevention and management among household contacts. Tailored interventions are needed to ensure information accessibility, especially for individuals living in suburban and rural areas.

## Data availability statement

The original contributions presented in the study are included in the article/Supplementary material, further inquiries can be directed to the corresponding author.

## Ethics statement

The studies involving humans were approved by the Ethics Committee of Suzhou Hospital of Integrated Traditional Chinese and Western Medicine (2022-001). The studies were conducted in accordance with the local legislation and institutional requirements. The participants provided their written informed consent to participate in this study.

## Author contributions

JW and YZ carried out the studies, participated in collecting data, and drafted the manuscript. PZ and XH performed the statistical analysis and participated in its design. FX participated in acquisition, analysis, or interpretation of data and draft the manuscript. All authors contributed to the article and approved the submitted version.
