# Supplementary material for: Knowledge, attitude, and practice toward tuberculosis prevention and management among household contacts in Suzhou Hospital, Jiangsu province, China
Source: Front Public Health. 2024 Mar 12;12:1249971. doi: 10.3389/fpubh.2024.1249971 (PMC10963489; doi:10.3389/fpubh.2024.1249971)
Supplement: Supplementary file 1 [file Table_1.DOCX]

| **Knowledge, attitude, and practice towards tuberculosis prevention and management among household contacts** | | | |
| --- | --- | --- | --- |
| **Part I Basic Information** | | | |
| **1.Your gender:** | | a. Male | b. Female |
| **2.Your age: years** | | | |
| **3.** **Marital status** | | a. Married  b. Unmarried/Divorced/Widowed | |
| **4.** **Residence** | | a. Rural  b. Urban  c. Suburban | |
| **5.** **Education level** | | a. Primary school and below  b. Middle school  c. High school/Technical secondary school  d. College and above | |
| **6.** **Work Status** | | a. Employed  b. Unemployed | |
| **7.** **Monthly family income per capita (CNY)** | | a. < 2000  b. 2000-4999  c. 5000-9999 | |
| **8.** **Medical insurance** | | a. Yes  b. No | |
| **9.** **Relationship with the patient** | | a. Spouse  b. Parents  c. Children/grandchildren and their spouses  d. Sibling | |
| **10.** **Disease duration of the patient** | | a. < 3 months  b. 3-6 months  c. 6 months-1 year  d. ≥ 1 year | |
| **11.** **Most recent smear result of the patient** | | a. Positive  b. Negative  c. Unclear | |
| **12.** **Treatment status of the patient** | | a. Initial treatment  b. Retreatment  c. Unknown | |

**Part II Knowledge of tuberculosis prevention and management**

| 1. Tuberculosis (TB) is a chronic infectious disease caused by Mycobacterium TB. | a. Correct | b. Wrong | c. Don't know |
| --- | --- | --- | --- |
| 2. TB is mainly transmitted through the respiratory tract, such as droplets and dust. | a. Correct | b. Wrong | c. Don't know |
| 3. People with latent TB infection are not TB patients and are not infectious. | a. Correct | b. Wrong | c. Don't know |
| 4. Coughing for longer than 2 weeks, or hemoptysis are common suspicious symptoms of TB, prompt consultation and treatment are needed. | a. Correct | b. Wrong | c. Don't know |
| 5. The regular medication treatment course for TB patients should be at least 6 months and be adjusted accordingly by considering the condition of TB and drug resistance. | a. Correct | b. Wrong | c. Don't know |
| 6. The patient can stop taking medications when the symptoms disappear during the treatment of TB. | a. Correct | b. Wrong | c. Don't know |
| The close contacts with negative results of TB screening test should be screened again after half a year and 1 year. | a. Correct | b. Wrong | c. Don't know |
| 8. BCG vaccination can prevent children from TB infection. | a. Correct | b. Wrong | c. Don't know |
| 9. Disinfection can be achieved by directly drying the articles used by patients in strong sunlight for half an hour. | a. Correct | b. Wrong | c. Don't know |
| 10. Boiling and high-pressure steam disinfection are the most effective methods to kill tuberculous bacteria, and should continue to boil for more than 10 minutes. | a. Correct | b. Wrong | c. Don't know |
| 11.Risk factors for TB: HIV infection, history of tuberculosis exposure, immune weakness, etc. | a. Know well | b. Partially know | c. Don't know |

**Part III Attitude to tuberculosis prevention and management**

| 1. TB is preventable and treatable. | a. Strongly agree | b. Agree | c. Neutral | d. Disagree | e. Strongly disagree |
| --- | --- | --- | --- | --- | --- |
| 2. You Are worried about being infected with TB. | a. Strongly agree | b. Agree | c. Neutral | d. Disagree | e. Strongly disagree |
| 3. The occurrence of TB patients in your family has a great impact on your life. | a. Strongly agree | b. Agree | c. Neutral | d. Disagree | e. Strongly disagree |
| 4. You are ashamed of having TB patients in your family. | a. Strongly agree | b. Agree | c. Neutral | d. Disagree | e. Strongly disagree |
| 5. You don't want others to know that there are TB patients in your family. | a. Strongly agree | b. Agree | c. Neutral | d. Disagree | e. Strongly disagree |
| 6. You can interact with tuberculosis patients with an attitude of equality and understanding. | a. Strongly agree | b. Agree | c. Neutral | d. Disagree | e. Strongly disagree |
| 7. TB patients and their household contacts will be discriminated by people around them. | a. Strongly agree | b. Agree | c. Neutral | d. Disagree | e. Strongly disagree |
| 8. You wish to get more information about TB prevention and treatment. | a. Strongly agree | b. Agree | c. Neutral | d. Disagree | e. Strongly disagree |
| 9. You are willing to receive preventive treatment if your doctor recommends it. | a. Strongly agree | b. Agree | c. Neutral | d. Disagree | e. Strongly disagree |
| 10. Would you agree to preventative treatment for close contacts in families with children under 5 years old? | a. Strongly agree | b. Agree | c. Neutral | d. Disagree | e. Strongly disagree |

**Part IV Practice on** **tuberculosis prevention and management**

| 1. Participants who continue to live in the same room with TB patient after diagnosis. | a. Yes | b. No |  |  |  |
| --- | --- | --- | --- | --- | --- |
| 2. Participants who continue to eat together with TB patient after diagnosis. | a. Yes | b. No |  |  |  |
| 3. Participants who went to hospital for TB screening after the patient in their household was diagnosed. | a. Yes | b. No |  |  |  |
| 4. Participants who received preventive treatment for TB. | a. Yes | b. No |  |  |  |
| 5.You will supervise the patient to take medications on time according to the doctor's advice. | a. Totally compliantly | b. Compliantly | c. Moderate | d. Not compliantly | e. Not compliantly at all |
| 6.You will try your best to avoid direct contact with patients. | a. Totally compliantly | b. Compliantly | c. Moderate | d. Not compliantly | e. Not compliantly at all |
| 7.You will remind patients and other family members to cover their mouths and noses with tissues when sneezing or coughing. | a. Totally compliantly | b. Compliantly | c. Moderate | d. Not compliantly | e. Not compliantly at all |
| 8.You will pay attention to opening windows for ventilation air, washing hands frequently, and taking active physical exercise. | a. Totally compliantly | b. Compliantly | c. Moderate | d. Not compliantly | e. Not compliantly at all |
| 9.You will take the initiative to learn about TB. | a. Totally compliantly | b. Compliantly | c. Moderate | d. Not compliantly | e. Not compliantly at all |
| 10. Access to the knowledge to in household contacts of tuberculosis patients. | a. Community outreach  b. Internet  c. Magazines and newspapers  d. TV and radio  e. Communication between friends  f. Introduction be medical staff  g. Otherways  h. Never cared about this information | | | | |
