# Supplementary material for: Knowledge, attitude, and practice toward tuberculosis prevention and management among household contacts in Suzhou Hospital, Jiangsu province, China
Source: Front Public Health. 2024 Mar 12;12:1249971. doi: 10.3389/fpubh.2024.1249971 (PMC10963489; doi:10.3389/fpubh.2024.1249971)
Supplement: Supplementary file 2 [file Table_2.docx]

**Supplementary Material**

Table S1 Differences in knowledge, attitude, and practice dimensions by residence among household contacts

| Items | Participants | | | |
| --- | --- | --- | --- | --- |
|  | Rural | Urban | Suburban | P |
| Basic knowledge on TB (K1-K4, K11), mean ± SD | 2.81 ± 0.81 | 3.71 ± 0.64 | 3.24 ± 0.82 | < 0.001 |
| TB treatment-related knowledge (K5-6), mean ± SD | 1.34 ± 0.88 | 1.76 ± 0.54 | 1.67 ± 0.66 | 0.002 |
| Contacts’ knowledge on TB prevention (K7-10), mean ± SD | 1.29 ± 1.03 | 2.43 ± 1.12 | 1.83 ± 1.13 | < 0.001 |
| TB is preventable and treatable (A1), mean ± SD | 2.66 ± 0.71 | 3.24 ± 0.54 | 2.97 ± 0.68 | < 0.001 |
| Stigma (A4-7), mean ± SD | 6.89 ± 2.15 | 7.29 ± 3.65 | 7.03 ± 1.75 | 0.405 |
| Would like to learn more knowledge (A8), mean ± SD | 2.78 ± 0.51 | 3.10 ± 0.54 | 2.88 ± 0.39 | 0.037 |
| Preventive treatment for children (A10), mean ± SD | 1.60 ± 0.54 | 2.05 ± 1.07 | 1.61 ± 0.52 | 0.338 |
| TB patients living alone (P1), mean ± SD | 2.63 ± 1.90 | 2.10 ± 2.05 | 3.37 ± 1.46 | < 0.001 |
| Eating with TB patients (P2), mean ± SD | 0.50 ± 1.32 | 2.86 ± 1.85 | 1.05 ± 1.76 | < 0.001 |
| Contact screening test (P3). mean ± SD | 3.70 ± 1.06 | 3.62 ± 1.20 | 3.91 ± 0.59 | 0.554 |
| Active learning (P9), mean ± SD | 2.50 ± 0.62 | 3.19 ± 0.81 | 2.65 ± 0.61 | < 0.001 |
